# Trapped in vicious cycles: unraveling the health experiences and needs of adults living with socioeconomic insecurity

**DOI:** 10.1186/s13690-024-01281-w

**Published:** 2024-04-16

**Authors:** Sanne E. Verra, Maartje P. Poelman, Andrea L. Mudd, John de Wit, Carlijn B.M. Kamphuis

**Affiliations:** 1 Department of Interdisciplinary Social Science, Utrecht University, Padualaan 14, 3584CH, 3584 CH Utrecht, the Netherlands; 2 Chair group Consumption and Healthy Lifestyles, Wageningen University & Research, Hollandseweg 1, 6706 KN Wageningen, the Netherlands

**Keywords:** Health inequities, Mental health, Health, Social security, Qualitative, Lay perspectives, Socioeconomic contexts, The Netherlands

## Abstract

**Background:**

This study explores the role of health in daily life and needs of Dutch adults (aged 25–49) experiencing one or more forms of socioeconomic insecurity stemming from their financial, housing, or employment situations.

**Methods:**

28 in-depth, semi-structured interviews were conducted in the Netherlands between October 2022 and February 2023. The interview guide included questions on participants’ socioeconomic situation, the role of health in their daily lives, their health-related and broader needs. Data was interpreted using inductive reflexive thematic analysis. An advisory board consisting of adults with lived experiences of socioeconomic insecurity were consulted at multiple stages of the study (recruitment, interview guide, interpretation of results).

**Results:**

Housing insecurity was widely experienced by participants. When asked about their financial situation, most participants expressed having no issues getting by, but later on, described vigorous efforts to minimize expenses. Participants’ narratives revealed four key themes in relation to the role of health in daily life and associated needs. Firstly, socioeconomic insecurity led to diminished control over life, which led to the disruption of routines. Secondly, experiencing socioeconomic insecurity compelled participants to prioritize stress reduction and mental health improvement through calming yet potentially damaging coping mechanisms. Thirdly, those who experienced little opportunity for improvement in their already long-lasting socioeconomic insecurity shared a sense of stagnation in life, which co-occurred with stagnation in unhealthy routines and diminished well-being. Fourthly, participants expressed the need for someone to speak with. This support may help participants regain control over their lives, identify opportunities for more socioeconomic security, and focus on increased health and well-being.

**Conclusions:**

This study sheds light on the challenges individuals face in dealing with socioeconomic insecurity, how it may affect their health, and their needs. Gaining perspective for improved socioeconomic security and having accessible professional support tailored to self-identified needs could have health-promoting effects for individuals living with socioeconomic insecurity. It is recommended to integrate professional support and assistance regarding social security into health policies and interventions. In future research, measures of financial strain should be adjusted to include the effort needed to get by.

**Table:** 

**Text box 1. Contributions to the Literature**
• Semi-structured interviews about health in daily life and needs were conducted.
• Socioeconomic insecurity made participants feel stagnant in life.
• Participants shared a need for a positive outlook on their socioeconomic situation.
• Accessible, professional help, tailored to self-identified needs is warranted.

## Background

Living in socioeconomic insecurity is strongly associated with poor health [1]. People who experience socioeconomic insecurity often live with structural disadvantages that may negatively impact their health, such as having fewer resources (e.g., lower incomes, less social capital), poorer living circumstances (e.g., poorer quality housing and neighborhoods with less green space and more noise and air pollution), and fewer opportunities (e.g., discrimination, unemployment) [2–4]. Socioeconomic insecurity can also lead to chronic stress, which has detrimental effects on mental health and well-being. Chronic stress has been hypothesized to place individuals in “survival mode”, in which there is a constant need to prioritize acute problems, such as coping with debts, making less energy available for less urgent goals, such as pursuing a healthy lifestyle [5–7].

Previous studies found that people in a lower socioeconomic position (SEP) were more likely to speak about their health in relation to structural and social determinants of health, whereas people in a higher SEP focused more on individual lifestyle [8–10]. People living in socioeconomic insecurity may have needs that are more salient for their health than striving for a healthy lifestyle [11–13]. Wink [14] found that priorities of those in socioeconomic insecurity were mainly related to reducing chronic stress by addressing what according to Maslow’s hierarchy of needs can be considered as basic needs [15], including financial security, housing, and safety. This has been acknowledged by other qualitative work among vulnerable populations in the Netherlands [16].

Due to several trends, the number of people experiencing socioeconomic insecurity has increased. First, inflation in the Netherlands was 10% higher in 2022 than in previous years, particularly due to increased prices for energy and food products [17]. This has placed many households at risk of financial insecurity [18]. Second, the current housing crisis in the Netherlands makes it challenging for people with low and middle incomes to find suitable housing [19]. Third, precarious employment contracts have become common in the Netherlands. In 2018, the proportion of the population in precarious temporary employment was 21.5%, which was double the Organization for Economic Cooperation and Development’s average [20]. People with lower incomes and educational levels are less likely to perceive their jobs as secure than people with higher incomes and educational levels [21]. Combined, these trends place large portions of the population at risk of socioeconomic insecurity.

Socioeconomic insecurity is estimated to be experienced by at least one in five Dutch adults [22] and has been suggested to disproportionately impact young adults [18, 23]. Currently, we lack understanding of how different forms of socioeconomic insecurity (related to income, housing, and employment) impact the role of health in adults’ daily lives and their needs, as most previous studies focused on specific, vulnerable target groups e.g., patients with chronic multimorbidities [24], older adults [25], unemployed adults [14, 26, 27], or people dealing with severe disadvantage such as homelessness [16]. Therefore, we interviewed adults in their prime working age (25–49 years old) who self-identified as experiencing some form of socioeconomic insecurity. We sought to understand how socioeconomic insecurity is connected to the role of health in their daily lives and needs. Insights can help inform policy makers and professionals about what to prioritize to improve the health of socioeconomically insecure adults.

## Methods

### Study design

We performed a qualitative study consisting of semi-structured, in-depth, one-on-one interviews. Ethical approval for the study was granted by the ethics committee of the Faculty of Social and Behavioural Sciences at Utrecht University (FSW FETC 24–0102).

### Consultations with advisory board

To include the perspectives of those with lived experiences of socioeconomic insecurity the researchers adopted a participatory approach [28] by incorporating feedback from members of the target population throughout the study. An advisory board, which was established in the department of Interdisciplinary Social Science at Utrecht University prior to the start of this study, was consulted. The advisory board consisted of ten Dutch adults who were living or had previously lived with socioeconomic insecurity. Members of this advisory board were recruited through a volunteering platform in Utrecht, the Netherlands. For this study, we consulted the advisory board during three 1.5 h long meetings, in which members provided input and advice on the interview guide (March 2022), the inclusion criteria and recruitment (November 2022), and the interpretation of the results (September 2023). Members received a compensation of €30 per meeting.

### Recruitment and data collection

Between October 2022 and February 2023, participants were recruited using purposive and snowball sampling. Inclusion criteria were being 25–49 years old, speaking Dutch, and self-identifying as having experience with socioeconomic insecurity. No minimum time period was attached to the criterium of experiencing socioeconomic insecurity. Socioeconomic insecurity was defined as experiencing insecurities regarding income, employment, or housing, in line with the definition of the Association of Dutch Municipalities (VNG) and the Association of Directors of Municipal Social Services (Divosa) [29].

The advisory board suggested several changes to our purposive sampling procedure. The advisory board requested the focus on people living in disadvantaged neighborhoods to be removed from recruitment materials, to prevent reducing one’s identity to the neighborhood they live in. The advisory board also suggested recruiting via key persons and by directly approaching people in public locations. Student assistants recruited 14 participants around shopping malls and the central railway station in Utrecht. People passing by were approached and informed that we aimed to gain a better understanding of people’s daily experiences and needs through interviews. If a person was interested, the inclusion criteria were explained, to establish the person’s eligibility. Nine participants were recruited via key figures (social employers) in the Hague, whose employees fit our inclusion criteria. Three participants were recruited via social media and two were recruited via snowball sampling. This resulted in a sample of 28 participants.

### Interview guide development

The interview guide was developed by SEV, MPP, and CBMK, and was adjusted based on consultation with the advisory board and insights from two pilot interviews. In the initial interview guide, respondents were asked about the meaning of health, then about health needs. The advisory board highlighted that this could give the impression of seeing participants as unhealthy and assuming that participants would desire making improvements to their health. In the revised version of the interview guide, respondents were first asked if they felt the need to improve anything about their current health, and, if so, what they would need to make those improvements.

A pilot interview revealed that the focus on health in the introduction seemed to steer the participant towards socially desirable answers, such as reiterating healthy lifestyle guidelines. This led to a further revision of the introduction and ordering of interview questions, which was changed to collecting detailed descriptions of participants’ context and daily life, while minimizing the focus on health in the first part of the interview. Participants were then asked about their definition of health, the role of health in their lives, health needs, and broader needs. A second pilot interview was conducted using the updated introduction and question ordering, which did not result in further changes to the interview guide. See the Web Appendix for the complete final interview guide.

### Interview procedures

All interviews were conducted by SEV. First, SEV introduced herself and the aim of the interview. Then, participants were informed that participation was entirely voluntary, that they had the right to refuse to answer any question, and that they could withdraw their participation at any point. Interviews began after obtaining informed consent.

23 interviews were conducted in person, either at the participants’ homes, Utrecht University, or public locations such as libraries or cafés. Four interviews were conducted by video call on Microsoft Teams, and one interview was conducted by phone. Interviews lasted between 30 and 77 min (mean and median: 53 min). After the interview, participants received a cash compensation of €24. After each interview, SEV took field notes and reflected on whether any personal interpretation could potentially bias the later analysis of the transcript, such as whether a participant reminded the interviewer of someone. Audio recordings of the interviews were transcribed verbatim by a professional transcription service. After checking transcripts for accuracy, the recordings were deleted. To avoid any potential identification of participants, all transcripts were pseudonymized and no ages or places of employment were reported.

### Data analysis

An inductive, reflexive thematic analysis (TA) was used, which aimed to uncover (latent) meanings in how socioeconomic insecurity shapes the daily experiences, health, and needs of participants. Data were analyzed for patterns of meaning following guidelines for inductive TA [30]. Data was analyzed by SEV in consultation with MPP and CBMK. The first transcript was analyzed by SEV, MPP, and CBMK, and the second and third transcripts were analyzed by SEV and either MPP or CMBK. The team discussed SEV’s interpretation of 14 more interviews. SEV drafted the themes, after which ALM read three transcripts to validate the interpretation of the themes and to check whether important themes were missed.

Six analysis steps were followed. The first five steps were inspired by Braun and Clarke’s (2006) guidelines for TA [30]: (1) reading and re-reading an entire transcript while taking initial notes, (2) coding of transcripts, which was done in Nvivo version 20, (3) developing and clustering emerging themes into a master theme list and writing memos about each individual interview. The memos enabled the analysis of each participants’ experiences in light of their socioeconomic circumstances and conceptualization of health, (4) reviewing the identified themes, and (5) write-up of the themes. In an additional sixth step, we presented advisory board members with the main themes and illustrative quotes, asked if they recognized the themes, if any important nuances were missed.

## Results

### Participant characteristics

Table 1 provides an overview of participants’ characteristics. The mean age of the participants was 35 years and participants had diverse educational levels. Half of the sample was not currently in paid employment. Some participants were on sick leave, stemming from issues such as burn-out or substance abuse. Most participants were born in the Netherlands, but many had international roots, including in Türkiye, Morocco, Surinam, Indonesia, or the Caribbean Netherlands.

**Table 1:** Overview of participants’ characteristics

	Number of participants (Total *N* = 28)
Primary education and lower secondary education	7
Upper secondary education	13
Tertiary education	8
Female	14
Male	14
In paid employment	14
In social employment	8
On sick leave due to illness	3
Unemployed, currently looking for work	1
Unemployed due to providing informal care	1
Full time student	1
Living alone	11
Living with children, not living with partner	2
Living with partner	2
Living with partner and child(ren)	5
Living with parents	3
Living with children and parents	2
Living with a housemate	3
Participant age	Mean: 35 (range 26–48)
Participant born in the Netherlands	22
Participant not born in the Netherlands	6 (all outside EU)
Both parents of participant born in the Netherlands	12
One or more parents of participant born outside of the Netherlands	14 (in EU: 2, outside EU: 12)
Unknown place of birth of parents	2

### Experiences of socioeconomic insecurity

Participants experienced varying severities and sources of socioeconomic insecurity. The different forms of insecurity were often interrelated and had positive or negative effects on health, as discussed below.

#### Housing-related insecurity

Around a quarter of participants seemed satisfied with their housing, but most participants found themselves in less than ideal housing situations. Many experienced their housing situation as completely out of their control and held little hope for improvement.

Many participants felt that they endlessly needed to rely on social housing waiting lists, as the private market was beyond their reach. This seemed particularly troublesome for some female participants who needed to find housing following breakups. For instance, since her divorce one year ago, Sarah time-shared an apartment with her former partner and lived with her parents part-time. Her former partner would soon terminate the rental contract of their apartment, forcing her to live with her parents full-time. Sarah described, “*The waiting time in [name village] is fifteen years, so when I got divorced, I immediately registered [on the waiting list]. So that will take fourteen more years.”* Interviewer: “*And do you have any idea what your options are?*” Sarah: “*No, I’m feeling really hopeless now. […] You don’t get priority [for social housing] unless there has been physical violence, and that is not the case. Fortunately, in a way, but on the other hand, I think, unfortunately…*” Desperation was also experienced by Nadine, who was a paying guest in the house of distant acquaintances. She explained that the lottery system of a social housing corporation offered some hope, but no sense of control, to those at the bottom of the social housing waiting lists. Participants felt forced to wait for severe setbacks, such as living on the street or becoming a victim of violence, to receive priority on the waiting list for social housing. However, even Koen, who had priority for social housing, needed to wait about four years for social housing. During this waiting time, he continued to live in an institution despite being capable of living on his own.

Although people in their social networks provided a roof to several participants, this also placed them in positions of feeling indefinitely stuck and not receiving priority for social housing. This was experienced by Laurens, who struggled living with his father: “*I’m still living in the same little room where I lived as a child, so at some point you really are just completely fed up with it. I find my living situation terrible.”* On the less extreme range of housing insecurity were participants who had been able to find secure housing, but struggled with their inability to live independently from housemates.

#### Finance-related insecurity

Many participants initially said that they had no issues getting by financially. However, they often later described intensively monitoring and minimizing their expenses by avoiding public transportation and social events, limiting gas and electricity use, sharing gym memberships, working (sometimes unofficial) side jobs, tracking and comparing prices of products across supermarkets, and seeking financial support for expensive purchases, such as eyeglasses. These expense-minimizing strategies were described by people with and without paid employment. That many participants did not perceive themselves as having financial issues could be linked to the sense of control that participants seemed to experience over their financial situations (often in contrast to their housing situation). Minimizing expenses meant forgoing what could be considered basic needs. However, participants seemed willing to spend much time and effort to maintain control over their finances. Stefan was one participant who expressed having no difficulties getting by, but, later in the interview, described going to great lengths to cover the costs of basic needs. When asked if he was able to get by financially, he confirmed that he was: “*Well, [making ends meet] didn’t happen for a very long time, but since I got help from [aid organization]… Yes, they assessed my financial situation, and since then, I have some money left over.”* Later on he said: “*Yes, I’m being a bit frugal.”* When the interviewer asked if he had the ability to sometimes do something fun or go for a drink, he replied: *“At the moment, I still need a good fridge and a good bed. So having fun, that is something for later.”* For many participants, meeting what could be considered basic needs was a struggle, and money needed to be saved to pay for basic items, such as a bed, heating, or public transportation.

#### Employment-related insecurity

Several participants mentioned an internal conflict between short-term and long-term employment security. Amy’s situation is a good illustration of this conflict. After deciding to quit her education for an underpaid but secure position, she had difficulties getting out of this employment situation due to a lack of energy, time, and money to pursue further education. This conflict was also experienced by self-employed participants, who had trouble finding a balance between less well paid, stable employment and better paid, self-employed yet insecure work. For other participants, their employment-related insecurity was interrelated with financial and housing struggles, since their wages did not enable them to afford better housing. Nearly all participants who experienced insecurity regarding their employment situation still held a positive outlook towards finding more secure employment in the future.

### The role of health in daily life and needs

We identified four main themes in relation to the role of health in daily life and associated needs of participants. The themes are related to: (1) how socioeconomic insecurity disrupted participants’ health routines, (2) how participants coped with socioeconomic insecurity through calming behaviors, (3) how participants felt stagnant in life and how this impacted their health, and (4) how having someone to talk to could help with socioeconomic insecurity and improve health.

#### Theme 1: socioeconomic insecurity disrupts health routines

Participants considered routines essential for their health. The most common examples of health routines that participants wished to stick to were being physically active through active transportation (e.g., walking, cycling) or sports, getting a sufficient amount of sleep (e.g., going to bed on time), cooking their own meals, and keeping up social participation (e.g., going to work or other out of home activities). Some consciously paid attention to their routines while experiencing socioeconomic challenges, as they considered routine a buffer against potential deterioration. This strategy was applied by Nadine, who shared, “*I never really sleep in. I haven’t done that from day one of the bankruptcy. Because I think: If I do that, if I stay in bed, it’s not going to turn out well.”*

However, for most participants, socioeconomic insecurity led to small, yet seemingly structural, disruptions to their health routines. Shamira noted: “*When I’m stressed, when I’m sad, then I do have the tendency to let everything go. You know? Even my healthy eating routines.”* Some participants explained that they either felt like having sufficient security allowed them to take care of their health routines, or they felt overwhelmed by their insecurity, which prevented them from sticking to health routines altogether. This was the case for Haiko, who described the role of health in his daily life as “*all or nothing*”. He described phases in his life where he either had stable routines and exercised a lot, or had no routine and spent his days on the couch. “*Once you’re in a good routine, then you’re good. Well, at the moment I am absolutely not [in a] good [routine].”* After losing his job, Haiko described getting into a “*downward spiral*” and becoming addicted to drugs. He explained: “*When I’ve been sick for a while or something like that. Then I’m completely out of my rhythm. Then, I also don’t feel like going anywhere or cycling, anything like that.”*

For many participants, a disruption of routines seemed to be an unavoidable consequence of socioeconomic insecurity. Nearly all respondents expressed a lack of and a need for more calmness to be able to restore their routines. This seemed to be particularly the case for those who experienced housing insecurity, like Sarah, who explained: “*I’m up to here with it. Yes, [the lack of] living space and just being able to establish some regularity in your own life*.” Calmness was the only need expressed by Jeroen: “*The only need I have is just to have peace of mind, just no nagging things on my mind”* Participants felt that especially improved housing security would positively influence their routines. When asked what housing would provide, Sarah replied, “*Well, regaining some calm and regularity and routine. And a life that I feel good about.*” Cheyenne said: “*Yes, calm. It would bring me calm. Freedom, control. That I decide what happens and what doesn’t happen, and what my house rules are.”* These insights highlight the importance of calm to stick to health routines.

#### Theme 2: coping with socioeconomic insecurity through calming behaviors

Participants expressed constantly feeling stressed due to their socioeconomic challenges, and participants often found themselves taking care of their health by engaging in various stress reducing and nerve-calming coping behaviors. For instance, Amy described: “*Yes, my new approach to health is to spend more time sitting outside, reading… To calm myself”.* Other positive coping behaviors participants turned to included physical exercise, mindfulness, journalling practices and expressing gratitude. However, most participants also turned to coping behaviors with potentially harmful effects in the long-term, such as smoking, eating pre-prepared meals, excessive gaming, and overeating.

Coping behaviors were often driven by a need for short-term calm. This was explained by Sarah, who picked up smoking following her divorce and the resulting housing insecurity. Despite being aware of the damaging health effects of smoking, she needed it to relax: *“I’m very aware of it. So, I try not to smoke too much… It’s not much, but these are small moments I take for myself from time to time. So that I can relax a bit.”* Koen, who was unemployed at the time of the study, explained that, in his situation, he needed to choose the “lesser evil” to improve his health: “*See, when you’re dealing with stress, you should smoke more in my opinion. […] Because, for me, the fear is that if I quit smoking, I’ll feel more stressed.”*

#### Theme 3: feeling stagnant in life and its potential impact on health

Due to difficulties escaping socioeconomic insecurities, several participants felt stagnant in life. For instance, Sarah described needing more housing security to be able to get her life back. Another participant, Nadine, described “*Of course, your life is upside down, so, in the beginning, you can’t do anything at all and you’re paralyzed. […] I just want to move on with my life. I feel like my life has been at a standstill for three years.”* When asked what she meant, she said: “*That you’ve lost everything and have become very isolated. Because the people around you also disappear. […] [Due to debts, ] I have had to leave everything behind. So, your life comes to a standstill.”* Feelings of stagnation were also reported by participants who explained how long-lasting their socioeconomic difficulties were. For instance, Ruben described feeling *“chained*” to his housing situation (living with his parents), and Jeroen mentioned having spent the last decade trying to get his life back on track. Richard explained: “*In a certain way, I have always been somewhat in survival mode”.* When asked if he has the feeling of still being in this state, he responds: “*Well, you have to make sure that you can manage it all, and… it’s never the case that you really have the peace of mind to take it all in for yourself… to really examine it.”* Being in survival mode compelled him to continue dealing with urgent struggles, without having the time, energy, or opportunities to take a step back to try to figure out how to overcome his socioeconomic insecurity.

Stagnation in relation to one’s socioeconomic situation, and the lack of opportunities to escape or improve these circumstances, might also be linked to feelings of stagnation in relation to health. Several participants described feeling stuck in sub-optimal health routines. Richard’s experiences illustrate this. He felt satisfied with his health, except for his social life and his physical fitness. When asked what he would need to improve this, Richards stated needing freedom, which he explained as the time and energy to make conscious improvements. “*I think that if I had more freedom and felt more energetic, I would also be more inclined to, for example,… tackle those weak points, That I would address those points more and that I would also look more closely into what else I want in life and devote more attention to those things.”* When asked if he could for instance shorten his 48-hour work week to have more free time, he explained feeling the constant pressure of his employment insecurity. Working fewer hours, he thought, could make his boss place him out of work, indicating difficulty of overcoming his sense of stagnation.

Many participants, when asked whether they would want to further improve their health, initially expressed ideas for behavior changes (e.g., engaging in more physical activity, cooking own meals). Later in the interview, intentions to change behavior did not seem strong. For example, Martijn said: “*I do know that those [microwavable] meals aren’t really very good. […] So, it’s better to cook. Yes, that might be one thing to improve, but I just find it very difficult. […] Yeah, I think it’s fine as it is at the moment.”* Daniela said: “*I also feel like: it’s all good the way it is now. So yes, I can improve a lot [about my healthy lifestyle], and it’s also fine the way it is now.”* Participants indicated that more socioeconomic security and feeling less stuck in life would allow them, in the words of two participants, “*to be human*”, and would allow them to engage in activities that could actually promote their health and well-being rather than maintaining the status quo. Unfortunately, for most participants, such as Tobias, “*actually being able to do something fun in the month”* felt unreachable as long as their socioeconomic insecurity persisted.

#### Theme 4: having someone to talk to could help with socioeconomic insecurity and improve health

In addition to the need for more socioeconomic security, one other need stood out. Many participants expressed the need to have someone to talk to, to be heard and seen. Talking to someone, such as a friend, social worker, or a psychologist, may help participants regain control over their lives and get a more positive outlook on their socioeconomic situation and well-being. Participants thought that talking to someone would help them to actively reflect on and find ways to improve their situation. They wanted to have conversations that focused on their self-expressed needs. Gideon shared: “*Someone really needs to come and shake me. To turn me upside down and say, ‘what’s really going on with you?’ And then I’ll talk about it, and I’ll realize, ‘Oh, you know, you’re stressing too much about a lot of things. Just go through your to-do list, set priorities, and then really work through them.’ That gives me peace of mind. Perspective. And then I see, ‘Oh, certain things are going well. Things that aren’t going so well, well, then you can improve them’.”*. Participants did not feel the need for suggestions from others or pre-determined treatment plans. Instead, they needed someone to help them identify their intrinsic goals and give them tools to solve problems and reach their goals.

Several participants had spoken to a professional at some point, such as a psychologist, social worker, or a coach, or had received help through their social employer. Most of these participants made use of (often incidental and temporary) free consultations, rather than more intensive and structural support. Other participants found talking to family members or friends helpful, and some found support through their religion or from their pets. To illustrate, Haiko, Jason, and Ming had reflective conversations with a professional, which allowed them to see their life and needs from different perspectives. This helped them find new solutions and get their lives back on track. Jason noted high societal costs as a drawback to this highly intensive form of help for those in socioeconomic insecurity but highlighted that it may be the only way to help people escape vicious cycles, saying: “*It’s a bit like having a financial debt buddy, but then for these [well-being] things, you know. That there’s someone who can personally assess the situation. And of course, that costs a lot of money, having someone who looks at each individual’s situation. But I really think it’s the only sustainable solution [to move forward in life].”*

## Discussion

This study explored the health, daily lives and needs of adults of prime working age (25–49 years old) living in a context of socioeconomic insecurity through qualitative interviews. The findings revealed that participants often seemed trapped in vicious cycles, where socioeconomic insecurity led to the disruption of health routines. Socioeconomic insecurities compelled participants to seek calming yet potentially damaging coping mechanisms. A sense of stagnation emerged in relation to participants’ socioeconomic situations and health routines, which likely reinforced damaging coping behaviors. Participants expressed the need for a more positive outlook on their socioeconomic situations and for talking to someone to reflect and regain control over their lives and well-being.

In line with other studies [11–14], the participants’ narratives illustrated the necessity of sustained effort and support to alleviate socioeconomic insecurity and the need to address the social determinants of health and their negative consequences on health and well-being. The social determinants of health can be defined as the socioeconomic, environmental, and political context individuals live in (yet have no or very little control over), such as housing, welfare, and employment conditions [31]. As long as the social determinants of health remained unaddressed, experiencing the rest, calm, and intentions needed to build health routines and make improvements to health and wellbeing seemed unlikely. Participants experienced feelings of stagnation concerning health along with unrealized health intentions. Participants often expressed needs such as calm, freedom, and quality of life, and noted that more socioeconomic security could help them meet their needs and maintain their preferred health routines.

The identified feelings of stagnation, in general, and in relation to health, share similarities with the findings of another Dutch study [25]. Among a predominantly older population in disadvantaged neighborhoods in the Netherlands, Berg et al. [25] identified fatalistic attitudes towards future socioeconomic situations and health outcomes. These could potentially result from prolonged stagnation, and could lead to being unable to actively improve health, and taking life (and illness) as it comes. It should be noted, as highlighted by our advisory board, that the inability to act on intentions related to one’s socioeconomic situation or health should not be interpreted as acceptance of the status quo.

There seems to be a mismatch between the expressed health needs of those who experience socioeconomic insecurity and traditional health promotion interventions, which are often solely targeted towards lifestyle improvements rather than towards the social determinants of health. This mismatch may explain the “*polite-yes-but-no responses*” of potential participants to intervention invitations, identified by Slagboom [13, p. 4]. Focusing solely on behavior change (e.g., nutrition improvement) is likely to be ineffective, especially when individuals find themselves in insecure socioeconomic contexts [8–10, 32]. Of the forms of socioeconomic insecurity, housing insecurity seemed to most severely influence health and well-being. Ensuring a sufficient supply of housing for individuals who currently face limited opportunities in the housing market, is essential. The waiting time for social housing in the Netherlands is currently seven years on average and can be as long as seventeen years [33]. Reconsidering who gets priority for social housing, as is already being done by some municipalities in the Netherlands [34], may reduce the threat of homelessness.

In addition to overcoming socioeconomic insecurity, participants believed that having someone to talk to could assist them in regaining control of their health and well-being. Although participants’ did not necessarily express the need to talk to a professional, professional help from for instance a psychologist or social worker is recommended given the severity of issues and the need for structurally embedded help. The need for professional support is in line with the findings of a previous study among parents living in disadvantaged neighborhoods [14]. In the Netherlands, professional coaching is more accessible to those with higher incomes than those with lower incomes [35]. In addition to removing financial barriers, it remains a challenge to ensure that this professional help is truly accessible. Especially those in insecure socioeconomic positions may experience stigma, preventing them from seeking (mental) support [36]. Members of our advisory board recommended connecting professional help to more accessible activities, such as a (free) haircut.

An important strength of this study is our collaboration with an advisory board consisting of people with lived experiences of socioeconomic insecurity throughout the research process. Since the study was conducted by a team of researchers in high socioeconomic positions, the collaboration with the advisory board made this study less prone to potential biases towards, for instance, the interpretation of health and health needs, stemming from having different socioeconomic perspectives. This collaboration increased the accuracy of our interpretation of the data. For instance, the advisory board members expressed concerns regarding our third theme, which we initially labelled ‘*Acceptance as a coping strategy for challenging socioeconomic situations*’. They explained that no one really accepts such an insecure situation, and the labeling and interpretation was seen as incorrect, leading us to return to the data. The rephrased theme was agreed upon by the board members. Another strength relates to our recruitment strategy, which involved proactively approaching people at shopping malls and through social employers. This led to a diverse sample in terms of ethnic background, age, gender, and educational level. Moreover, attention was paid to avoid potential social desirability towards health in the participants’ answers.

A potential limitation of our study is that the need to talk to someone may have arisen through selection bias since all participants were open to being interviewed and to talking about their situations. Due to the stigma attached to socioeconomic insecurity, it is possible that participants held back on some of their negative experiences. Although this study attempts to draw some causal connections (for instance between disrupted health routines, health decline, and socioeconomic insecurity), this study cannot provide evidence on causality.

To get a deeper understanding of vicious cycles between socioeconomic insecurity and health, and of possibilities to escape these cycles, future research should explore the longitudinal impact of health on the daily lives of those in socioeconomic insecurity and should study potential ways to break these vicious cycles. We highly recommend working with people with lived experiences with the research topic.

Lastly, it seems important for future research to reassess the measurement of financial strain and financial scarcity. Surveys often include questions asking respondents if they have difficulties making ends meet [e.g., 18, 37], using statements such as “*I often don’t have enough money*”, and “*I experience little control over my financial situation*”. This study highlights a crucial distinction: instead of *experiencing* financial trouble, participants expressed *going through trouble* to get by. Most of our participants, while going to great lengths to make ends meet, did not self-identify as being financially insecure. This nuance was also raised by Platzer et al. [9] among a Dutch sample. Continuing to use the current measures might lead to an underestimation of the influence of financial strain on health outcomes and may explain the widespread experience of financial strain across all income levels [38].

## Conclusions

In conclusion, this study sheds light on the experiences of prime working age adults facing socioeconomic insecurity in the Netherlands. The experiences of participants, who felt trapped in insecure socioeconomic positions, often despite working many hours, highlight the limited social mobility and opportunities for more security. The experiences of participants underline a need for policies that address the social determinants of health, most importantly through improved access to suitable housing. Furthermore, participants emphasized the need for someone to talk to. Talking to someone was perceived as a valuable way for participants to help reflect on and regain some control over their lives, reach a sense of calm, and enhance their overall health and well-being. Lastly, findings suggest that the re-evaluation of the measurement of financial strain in surveys is needed to more accurately represent people’s experiences.

## Data Availability

The data used and analyzed in the current study are available from the corresponding author on reasonable request.

## Abbreviations

SEP: Socioeconomic position
